# Kidney Transplantation in a Patient Affected by Sickle Cell Trait: A Case Report and State-of-the-Art Review

**DOI:** 10.7759/cureus.33400

**Published:** 2023-01-05

**Authors:** Daniela Picciotto, Elisabetta Bussalino, Francesca Viazzi, Ernesto Paoletti, Pasquale Esposito

**Affiliations:** 1 Unit of Nephrology, Dialysis and Transplantation, Istituti di Ricovero e Cura a Carattere Scientifico (IRCCS) Ospedale Policlinico San Martino, Genova, ITA; 2 Department of Internal Medicine, University of Genova, Genova, ITA

**Keywords:** immunological risk, infectious complications, sickle cell anemia, hereditary nephropathies, hemoglobinopathy, sickle hemoglobin, sickle cell trait, kidney transplantation

## Abstract

Chronic kidney disease (CKD) is a common feature of sickle cell disease (SCD). The awareness of the clinical presentation and renal involvement in patients affected by hemoglobinopathies is greatly needed. Patient management is particularly complex, especially with kidney transplantation. We, therefore, report the case of a 56-year-old patient affected by sickle cell trait who underwent kidney transplantation. This case will underline all the various challenges the nephrologist must face in this clinical setting and their management.

## Introduction

Sickle cell disease (SCD) is a genetic homozygotic inherited hemoglobinopathy due to a substitution of valine for glutamine at the sixth codon of the beta-globin chain (hemoglobin [Hb]SS genotype). The disorder inherited in heterozygous form is named sickle cell trait (HbSA or sickle hemoglobin [HbS] variants).

Renal disease is a common feature of SCD, and chronic kidney disease (CKD) develops in nearly 50% of the patients and is associated with poor prognosis and high mortality rates [1]. Kidney damage may present because of a vaso-occlusive crisis induced by red blood cell sickling secondary to hypoxic, ischemic, and inflammatory triggers. In this condition, the increased blood viscosity deranges the renal microcirculation leading to glomerular and tubular ischemia, interstitial fibrosis, tubular atrophy, and glomerulosclerosis. Moreover, in SCD patients, various types of glomerulopathies can be observed, and more rarely, obstructive nephropathy due to papillary necrosis caused by medullary ischemia [2-4]. Of note, sickle cell trait carriers are reported to have a 1.5 to two-fold increased risk of developing CKD, too [5].

In a globalized world, the awareness of the clinical presentation and renal involvement in patients affected by blood disorders and hemoglobinopathies is much needed. Indeed, the management of CKD in these patients is particularly complex, especially kidney transplantation, with still many unresolved issues [6].

Here, we report the case of a patient affected by sickle cell trait, presenting a challenge to clinical management from CKD progressing to kidney transplantation.

## Case presentation

Our patient is a 56-year-old woman first diagnosed with autosomal dominant polycystic kidney disease (ADPKD). The diagnosis was imaging-based and reached after several episodes of renal colic associated with macrohematuria without stone expulsion. Her medical history was otherwise uneventful except for hypertension and migraine. She progressively developed end-stage renal disease (ESRD), and hemodialysis was started at our unit in 2010. Soon afterward, she underwent left and right nephrectomies for hemorrhagic and complicated renal cysts.

In the following years, her clinical condition deteriorated. She presented a severe cardiovascular impairment characterized by a dilated cardiomyopathy with low ejection fraction and several episodes of pulmonary edema associated with a hypertensive crisis.

Moreover, she often complained about chronic diffuse pain and migraine attacks. Most importantly, she manifested, especially during hemodialysis, frequent seizures requiring antiepileptic medications.

Because of the persistence of transfusion-dependent anemia, only partially responsive to erythropoietin administration, we suspected an underlying hemoglobinopathy.

In February 2017, sickle cell trait was diagnosed with evidence of a concentration of sickle hemoglobin (HbS) of 31.5%. She, therefore, started treatment with hydroxyurea to augment HbF concentration and modulate erythrocyte rheology. Unfortunately, this medication was soon interrupted due to leucopenia. Since April 2019, erythrocytapheresis sessions (an automated blood exchange procedure which consists in removing red blood cells containing HbS and replacing them with normal blood cells) were planned every 45 days to maintain HbS under the 20% threshold. The patient's clinical condition subsequently improved, the number of seizures and painful crises reduced, and most importantly, cardiac function normalized. Then, she was included on the kidney transplant waiting list. Because of polytransfusions, the patient had a high immunological risk with a maximum panel reactive antibody (PRA) score of 90%.

In early June 2020, the patient underwent kidney transplantation. The deceased donor was a 62-year-old male. Creatinine at the time of explant was 0.9 mg/dl, and proteinuria was 0.15 mg/dl. The number of mismatches was four (one in human leukocyte antigen [HLA]-A, two in HLA-B, and one in HLA-DR). At the transplantation, the PRA score of the recipient was 0%. Donor-specific anti-HLA antibodies (DSA) were undetectable. We applied our high immunological risk protocol. It included induction with basiliximab, anti-thymocyte globulin (ATG), and high-dose intravenous methylprednisolone with subsequent tapering, followed by maintenance immunosuppression with tacrolimus and mycophenolate mofetil. HbS concentration at the time of transplantation was 26%, and the blood count revealed stable pancytopenia with hemoglobin (Hb) of 9.1 g/dl, white blood cell count (WBC) of 3540/mm^3^, and platelet count (PLT) of 115.000/mm^3^. Before transplantation, the patient underwent two blood transfusions and oxygen support to maintain an oxygen blood saturation of at least 96-98%. After transplantation, we observed a progressive improvement in graft function. Creatinine at discharge was 1 mg/dl. During the immediate post-transplantation period, she developed *Escherichia coli* (E. coli) and *Enterococcus faecium* urosepsis treated with antibiotics. Multiple blood transfusions were required because of persistent anemia with Hb <8 g/dl. During hospitalization, we regularly determined HbS concentration, which was found to be lower than 20%. In December 2020, she contracted a paucisymptomatic severe acute respiratory syndrome coronavirus 2 (Sars-Cov-2) infection. The subsequent hospitalization was complicated by a left ischemic stroke; the Hb values at the time of the neurological event ranged between 8.0 and 8.5g/dl without erythropoiesis-stimulating agents (ESA) treatment, and HbS was 22%. In the following months, the patient developed frequent and relapsing E. coli urinary tract infections and urosepsis. A nuclear magnetic resonance (NMR) imaging of the abdomen performed in July 2021 showed signs of chronic pyelonephritis in the graft. Subsequently, we decided to cease oral therapy with steroids and substitute tacrolimus with cyclosporin. In December 2021, after another episode of E. coli-mediated urosepsis, she underwent an allograft biopsy due to a progressive worsening of her renal function with creatinine values of 1.9 mg/dl despite appropriate antibiotic therapy and sterile urine. A histological diagnosis of an acute active antibody-mediated rejection (ABMR) associated with a borderline T-cell-mediated rejection (TCMR) was made. DSA testing was negative. Therefore, the patient was treated with steroid bolus and plasmapheresis followed by an infusion of 2 g/kg of intravenous immunoglobulins. Moreover, tacrolimus, instead of cyclosporin, and oral prednisone in association with mycophenolate mofetil have been reintroduced as maintenance immunosuppressive therapy. Her kidney function at the time of discharge was stable, with creatinine values of 1.6 mg/dl, HbS level of 28.6%, and Hb between 8 and 9 g/dl.

In August 2022, the patient contracted Sars-Cov-2 infection again, characterized by bilateral interstitial pneumonia and respiratory failure. The patient was treated with antiviral agents with subsequent clinical improvement. Nevertheless, the hospitalization was complicated by seizures and the need for blood transfusions, which, however, allowed to maintain Hbs values between 19 and 16%.

At the last follow-up in October 2022, the patient was in stable clinical condition. Creatinine was 1.8 mg/dl, proteinuria was 0.15 g/24 hours, Hb was 11 g/dl, and HbS was 33.5% (an erytrocytapheresis session was planned to be repeated soon afterward). Asymptomatic bacteriuria was detected in the urine culture test.

## Discussion

Even though sickle cell glomerulopathy and renal involvement are more frequent in patients with homozygous HbSS, renal abnormalities are also associated with the sickle cell trait, constituting an additional risk factor in patients with CKD from other causes [7]. In the clinical case reported here, the principal cause of ESRD was ADPKD, while sickle cell trait was diagnosed several years after beginning renal replacement therapy. However, we cannot exclude concomitant damage in the native polycystic kidneys caused by her hemoglobinopathy. Interestingly, a study by Yium et al. reproduced evidence that a cohort of ADPKD Black patients with sickle cell trait showed an augmented incidence of ESRD compared to black ADPKD patients without sickle hemoglobin. The authors suggested that medullary ischemia may be the causative factor of accelerated cystogenesis [8].

Moreover, our case may be of particular interest as it includes all the challenges and features that characterize the management of kidney transplantation in a patient affected by SCD or sickle cell trait. Regarding the perioperative management of our transplant recipient, we decided to apply some evidence-based suggestions reported in the literature [9,4], which were confirmed by recent anesthesiological guidelines [10]. These suggest the maintenance of a pre-operative Hb of 10 g/dL and intense postoperative care in SCD patients, including the administration of intravenous fluids to avoid volume depletion and reduce blood viscosity. Additionally, the maintenance of normothermic conditions, as well as peripheral oxygen saturation of at least 96%, are of utmost importance.

Notably, the subsequent course of our patient confirms previous observations in SCD, namely the high occurrence of infections because of a deranged immunological system. In a recent study by Gérardin et al., pyelonephritis was found to be the most frequent complication in transplanted SCD patients [11]. The consequent need for minimizing immunosuppression surely represents a struggle for clinicians because of the high immunological risk often present in these patients due to polytransfusions. Because of the recurrent urinary tract infections, we decided to minimize the maintenance immunosuppression regimen. Unfortunately, this choice was followed by the occurrence of acute ABMR, as well as a borderline TCMR, as proof of the extremely insidious decision-making process.

So, the management of this patient posed several presumptive extra-renal complications related to the presence of sickle hemoglobin, as well as many vexing issues regarding the medical management of the kidney transplantation itself (see Table 1).

**Table 1:** Key steps of kidney transplantation management in patients affected by sickle cell disease or trait SCD - sickle cell disease; HbS -  sickle hemoglobin; DSA - donor-specific anti-HLA antibodies; HLA - human leukocyte antigen

Key steps	SCD related risks	Management and hints	
Access to the kidney transplant waiting list	Delayed access to the waiting list due to systemic complications	Hematological referral + monitoring of HbS; treatment with hydroxyurea and/or erythrocytapheresis sessions aiming at HbS <20%; multidisciplinary management	
Access to kidney transplantation	Long waiting time due to a high immunological score	Hb levels to be maintained between 8 and 10g /dl with erythropoiesis-stimulating agents avoiding hemotransfusions	
Perioperative transplant management	Increased cardiovascular morbidity; risk of acute chest syndrome after surgery	Pre-operative Hb of 10 g/dL; intravenous fluids to avoid dehydration and reduce blood viscosity; ensure normothermic conditions; peripheral oxygen saturation of at least 96%	
Follow-up	Infectious complications	Minimization of maintenance immunosuppression; if recurrent urinary tract infections, consider long-term antibiotic prophylaxis supplements for urinary tract infections, e.g., cranberry extract; D Mannose	
	High immunological risk	Strict monitoring of proteinuria and de novo DSA development; eventual protocol allograft biopsies	
	Relapse or de novo sickle cell nephropathy	Strict monitoring of proteinuria; treatment with hydroxyurea and/or erythrocytapheresis sessions aiming at HbS <20%	

From now on, the program for our patient is to implement a strict follow-up continuing regular erythrocytapheresis sessions planned according to HbS values. Noteworthy, this treatment has been recently correlated with improved patient and graft survival [12]. Finally, it should be highlighted that the advantage of kidney transplantation in SCD patients is still controversial. An analysis of the Organ Procurement and Transplantation Network (OPTN)/United Network for Organ Sharing (UNOS) 2000-2019 database showed that patient and graft survival of sickle cell patients are worse than those of other recipients [13]. On the other hand, mortality among SCD patients on hemodialysis exceeds that of patients without hemoglobinopathies, while renal transplantation seems effective in reducing mortality [14].

## Conclusions

So, these data suggest that despite the high risk of failure and the difficult management, kidney transplantation may have beneficial effects on the survival of sickle cell patients. Transplantation programs should be, therefore, encouraged.

In conclusion, hemoglobinopathies represent an underrecognized cause of renal disease characterized by insidious clinical management. Kidney transplantation in patients affected by SCD or sickle cell trait presents a difficult challenge because of the frequent perioperative complications and the coexistence of both high immunological and infectious risks, as demonstrated by our clinical case. Nevertheless, the survival benefit offered by kidney transplantation, together with the presumable improvement of quality of life, urgently imposes the development of dedicated strategies and guidelines.
